# Changes in retinal vascular bifurcation in eyes with myopia

**DOI:** 10.1186/s12886-022-02629-y

**Published:** 2022-10-21

**Authors:** Caixia Sun, Tingli Chen, Jing Cong, Xinyuan Wu, Jing Wang, Yuanzhi Yuan

**Affiliations:** 1 Department of Ophthalmology, Zhongshan Hospital, Fudan University, 200032 Shanghai, China; 2 Department of Ophthalmology, Huadong Sanatorium, Wuxi, Jiangsu Province China; 3 Department of Ophthalmology, Zhongshan Hospital (Xiamen), Fudan University, Xiamen, Fujian Province China; 4 Center for Evidence-based Medicine, Fudan University, Shanghai, China

## Abstract

**Objective:**

To evaluate the effect of myopia on retinal vascular bifurcation.

**Methods:**

A cross-sectional study that retrospectively analyzed the fundus photographs and clinical data of 493 people who participated in routine physical examinations in Huadong Sanatorium. One eye of each subject was included in the analysis. Retinal vascular bifurcation measurements were extracted by using a validated computer program. One-way ANOVA and analysis of covariance were performed to compare the measurements across high myopia, low to moderate myopia, and non-myopia groups.

**Results:**

The mean age was 41.83 ± 10.43 years and 63.49% were women. The mean spherical equivalent refraction (SER) was − 4.59 ± 3.07 D. Ninety-nine (20.08%) eyes met the definition of high myopia (SER ≤ -6.0 D), along with 234 (47.46%) low to moderate myopia (-6.0 D < SER <-0.5 D), and 160 (32.45%) non-myopia (SER ≥ -0.5 D). The differences in the arteriolar branching angle, venular branching coefficient, venular asymmetry ratio, venular angular asymmetry, and venular junctional exponent among the three groups remained significant (*p* < 0.05) after multivariate adjustment. Pairwise comparisons showed arteriolar branching angle and venular angular asymmetry in high myopia were significantly lower than low to moderate myopia (*p* < 0.001, *p* = 0.014 respectively) and non-myopia (*p* = 0.007, *p* = 0.048 respectively). Venular asymmetry ratio and venular branching coefficient in high myopia were significantly higher than low to moderate myopia (*p* = 0.029, *p* = 0.001 respectively) and non-myopia (*p* = 0.041, *p* = 0.043 respectively). There was a significant difference in venular junctional exponent between high myopia and low to moderate myopia (*p* = 0.031).

**Conclusion:**

The vascular bifurcation differs in dependence on the myopic refractive error and a significant increase in the difference can be observed in high myopic eyes.

## Introduction

A healthy vascular network is essential for efficient blood transport to various parts of the body, and damage to the network may cause ischemia, leakage, and other pathologic conditions which then lead to many diseases. The retinal vascular network can be directly observed in vivo and has therefore been studied widely in expectation of finding predictors of various diseases. Studies have shown that the retinal vascular network geometry changes are an important sign of vascular damage, which are associated with a variety of systemic and ocular diseases, such as hypertension [1], diabetes [2], cerebral small vessel disease [3], dementia [4], diabetic retinopathy [5], and glaucoma [6].

In myopic eyes, the expansion of sclera stretches and thins the underlying choroid and retina [7], which is responsible for the myopia-related retinal vascular alterations, for instance, narrower arterioles and venules [8–10], higher length to diameter ratios of retinal arterioles [11], straighter arterioles [9], and decreased vascular fractal dimension [12]. However, the relationship between myopia and vascular bifurcation remains controversial. Patton et al. [8] found no association of axial length (AL) with the angles at vessel bifurcations and junctional exponents in a small pseudophakic population. However, Lim et al. [9] demonstrated in a middle-aged and older Malay population that increased myopic refractive errors and longer AL were associated with more acute branching angles in arterioles, and increased branching coefficients (BC; quotient of the area of the branch and trunk vessels, Table 1) in both arterioles and venules. The discrepancy in the results may be due in part to potential or residual confounding. Aging, cardiovascular disease, cardio-active medications, smoking, etc. are known to be associated with the retinal vascular geometry, and are not possibly fully adjusted in a relatively older and heterogeneous study population, which in turn hinders our understanding of the relationship between myopia and retinal vascular geometry. In addition, if the bifurcation parameters change with the myopic elongation of the globe, a dose-response effect should be observed, which however has not been carefully studied yet.

Therefore, we conducted a study investigating the differences in retinal vascular bifurcation parameters among high myopia, low to moderate myopia, and non-myopia in an otherwise healthy and relatively young population.

## Methods

In this cross-sectional study, we retrospectively reviewed the fundus photographs and clinical data of 1100 people who participated in routine physical examinations in Huadong Sanatorium from June 2018 to December 2018. The present study was conducted in accordance with the ethical principles of the Declaration of Helsinki on medical research. The retrospective review of participants’ records and the waiver of consent were approved by the Medical Ethics Committee of Huadong Sanatorium. Subjects with the following conditions were excluded from this study: (1) History of cataract surgery, aphakic or pseudophakic, and self-reported refractive surgery in the selected eye (n = 0); (2) Diabetic retinopathy and other retinal vascular diseases in the selected eye (n = 2); (3) History of smoking, hypertension (medical history or meet the diagnostic criteria of hypertension[systolic blood pressure (SBP) ≥ 140 mmHg and/or diastolic blood pressure (DBP) ≥ 90 mmHg] [13]), diabetes(medical history or glycosylated hemoglobin ≥ 6.5 mmol/l [14]) (n = 531); (4) Fundus images of poor quality, including those due to media opacities, small size of pupil or images out of focus (n = 74). Of the 1100 available people, 493 people were included in this study.

Each subject underwent a comprehensive ocular examination during physical examination, including pupillary examination, anterior segment examination, and complete retinal evaluation. Noncycloplegic autorefraction was performed using an auto-refractometer (Topcon KR-8900, Japan). Intraocular pressure was measured in mm Hg with a non-contact tonometer (Topcon CT-80 A, Japan). Digital fundus photography was taken by using a 45° digital retinal camera (Topcon-NW300; Japan) without mydriasis. Two retinal images of each eye were obtained, one centered at the optic disc and another centered at the fovea.

For each subject, we selected one optic disc-centered photograph from the eye with better photographic quality to perform measurement (when the photographic quality of both eyes was similar, the right eye was selected for analysis). The refractive state of this eye determined the refractive group that the subject was assigned to. Spherical equivalent refraction (SER) was calculated as the sum of the sphere power and half of the cylinder power, and the unit is diopter (D). Refractive errors were defined as high myopia (SER ≤-6.0 D) [15], low to moderate myopia ( -6.0 D < SER <-0.5 D) [15], and non-myopia (SER ≥ -0.5 D).

## Measurements of retinal vascular bifurcation parameters

**Fig. 1:**
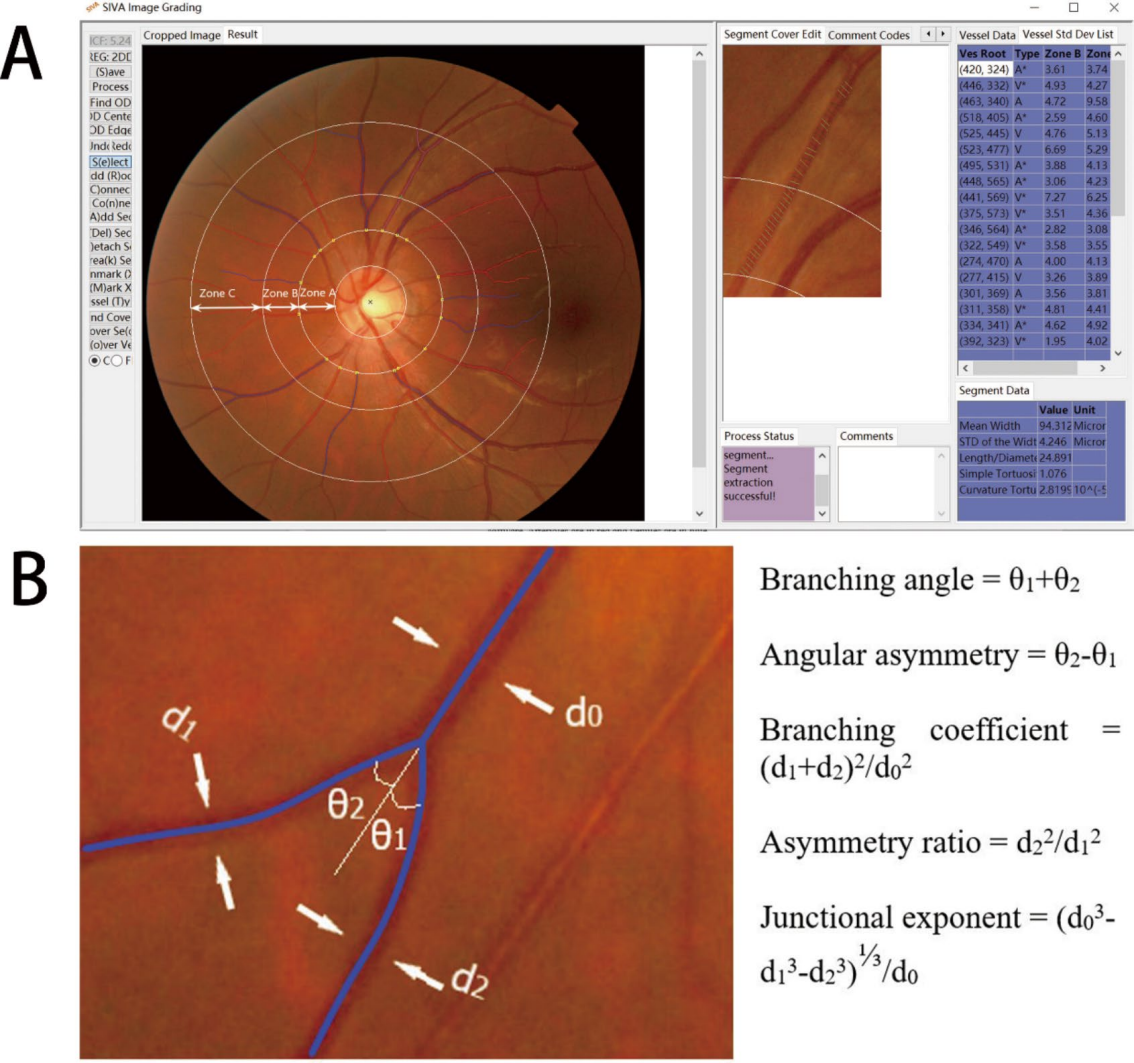
(A) Retinal vascular geometric variables assessed quantitatively by the Singapore I Vessel Assessment software. Arterioles are in red and venules are in blue. (B) At each bifurcation, the width of the trunk vessel (d_0_) and its two branching vessels (d_1_ and d_2_, d_1_ > d_2_) are measured, as well as the branching angle (θ_1_ and θ_2_, θ_1_ < θ_2_)

For each retinal photograph, a trained grader (SCX), masked to participants’ identities, used a semi-automated computer-assisted program (Singapore I Vessel Assessment [SIVA], version 4.0; National University of Singapore, Singapore) to quantitatively measure a series of retinal vascular bifurcation parameters. During image grading, SIVA divides the retina into three zones with reference to the optic disc location and size. Zone A, B, C are standardized and defined as the region from the disc margin to 1/2-disc diameter from the disc margin, 1/2-disc diameter to 1 disc diameter from the disc margin, and 1 disc diameter to 2-disc diameter from the disc margin, respectively (Fig. 1 A). Retinal vessel geometric parameters are measured and calculated based on the retinal vessels located in Zone B and C as measurements in these zones are more stable from curvature of the eye. A description of retinal bifurcation parameters is listed in Table 1 and depicted in Fig. 1B. Previous studies reported that SIVA produces repeatable measurements of the retinal vasculature in former preterm and term children and in adults; intra-observer and interobserver reproducibility were higher than 66% for vascular geometry [16].

**Table 1:** Description of the retinal vascular bifurcation parameters measured for each retinal photograph

Parameter	Abbreviation	Description
Branching angle	BA	Defined as the first angle subtended between two daughter vessels at each vascular bifurcation.
Branching coefficient	BC	Branching coefficient reflects the ratio between the diameters of the parent vessel and the diameters of its branches. Defined as the summed square of the mean vessel widths of each branch or daughter vessel divided by the square of the mean width of the parent vessel.
Asymmetry ratio	AF	Defined as the ratio of the square of the width of two branching vessel.
Angular asymmetry	AA	Defined as the difference between two daughter angles.
Junctional exponent	JE	Junctional exponent provides an index of caliber sizes of two daughter vessels relative to the parent vessel and is considered to represent an optimality state of microvascular networks [17]. It has been proposed that in an optimal state, the value of junctional exponent is three [17].

### Statistical analysis

Statistical analysis was performed using SPSS V.23.0 (SPSS Inc., Chicago, IL). The branching coefficient, branching angle, asymmetry ratio, and angular asymmetry were normally distributed among participants, as well as the logarithmically transformed junctional exponent. Descriptive statistics were presented as mean and standard deviation (SD) for normally distributed data.

Chi-square test (categorical variables) and one-way ANOVA (normally distributed continuous variables) were used to perform comparisons among different refractive error statuses. The multivariable-adjusted model was tested for each dependent variable with correction for confounding variables in analysis of covariance (ANCOVA). Age [18], glycosylated hemoglobin [19] (HbA1c), mean arterial pressure [1] (MAP, calculated as 1/3SBP + 2/3DBP [20]), body mass index [21] (BMI, calculated as weight in kilograms divided by the square of height in meters) and intraocular pressure [6] (IOP) were known to influence retinal vascular bifurcation parameters and included as covariates. In order to further identify which particular differences between pairs were significant, we used pairwise comparison to explore differences among three groups. Moreover, we used complete case analysis to deal with a small number of missing data on HbA1c (n = 19), BMI(n = 23), IOP(n = 7) and MAP (n = 4). The missing data was considered as missing completely at random. One-way ANOVA was performed after missing values were removed. Participants with missing data were excluded from analysis of covariance and pairwise comparisons.

## Results

Retinal photographs of unilateral eyes from 493 people were included in this study. The mean age was 41.83 ± 10.43 year-old (ranged from 17 to 75 year-old ), with 180 men (36.51%) and 313 women (63.49%). The mean SER was − 4.59 ± 3.07 D. Ninety-nine (20.08%) eyes met the definition of high myopia (ranged from − 16.13 D to -6.00 D), along with 234 (47.46%) low to moderate myopia (ranged from − 5.88 D to -0.63 D), and 160 (32.45%) non-myopia (ranged from − 0.50 D to + 6.13 D). Systemic demographics and retinal vascular bifurcation parameters of the participants grouping by the refractive error status are summarized in Table 2. In general, myopic subjects were younger, and with lower HbA1c. Mean arteriolar branching angle, arteriolar branching coefficient, venular branching coefficient, venular asymmetry ratio, venular angular asymmetry, and arteriolar junctional exponent were significantly different among the three different refractive error status groups.

**Table 2:** Clinical characteristics and retinal vascular bifurcation measurements, by refractive error status

Parameter	High myopia	Low to moderate myopia	Non-myopia	*p* value
Number	99	234	160	
Age (years)	38.67 (10.56)	39.35 (8.95)	45.48 (10.70)	< 0.001*
Female, n (%)	62 (62.62%)	142 (60.68%)	109 (68.13%)	0.486
Spherical equivalent (D)	-8.62 (2.23)	-2.99 (1.44)	0.26 (0.92)	< 0.001*
Intraocular pressure (mmHg)	15.37 (2.76)	15.40 (2.58)	15.14 (2.46)	0.750
Body mass index (kg/m^2^)	23.33 (3.78)	22.48 (2.64)	23.11 (2.84)	0.208
Glycosylated hemoglobin (%)	5.30 (0.35)	5.24 (0.28)	5.41 (0.31)	< 0.001*
Total cholesterol (mmol/L)	4.96 (0.68)	4.95 (0.83)	5.05 (0.81)	0.299
Systolic Blood Pressure (mmHg)	115.01 (13.89)	113.19 (12.35)	115.19 (11.16)	0.481
Diastolic Blood Pressure (mmHg)	71.04 (9.86)	69.38 (8.36)	69.67 (8.68)	0.398
Arteriolar branching angle (degree)	70.11 (11.73)	76.59 (11.41)	75.30 (10.66)	< 0.001*
Venular branching angle (degree)	74.51 (12.69)	76.61 (9.94)	75.97 (10.70)	0.277
Arteriolar branching coefficient	1.48 (0.21)	1.41 (0.18)	1.47 (0.19)	0.001*
Venular branching coefficient	1.31 (0.18)	1.26 (0.14)	1.27 (0.15)	0.014*
Arteriolar asymmetry ratio	0.82 (0.07)	0.82 (0.06)	0.81 (0.07)	0.112
Venular asymmetry ratio	0.74 (0.10)	0.71 (0.10)	0.72 (0.10)	0.041*
Arteriolar angular asymmetry (degree)	30.28 (13.52)	33.76 (11.44)	32.56 (11.25)	0.051
Venular angular asymmetry (degree)	37.21 (13.55)	41.39 (13.41)	41.02 (12.46)	0.026*
Log (arteriolar junctional exponent)	0.41 (0.06)	0.43 (0.05)	0.41 (0.05)	0.002*
Log (venular junctional exponent)	0.45 (0.06)	0.46 (0.05)	0.46 (0.05)	0.224

All data were expressed as mean (SD) or number (percentages), as appropriate. Chi-square test (categorical variables) and one-way ANOVA (normally distributed continuous variables) were used to perform comparisons. P-values were corrected for multiple comparisons using the Bonferroni method

*Statistical difference (*p* < 0.05) among participants with different refractive error status

**Fig. 2:**
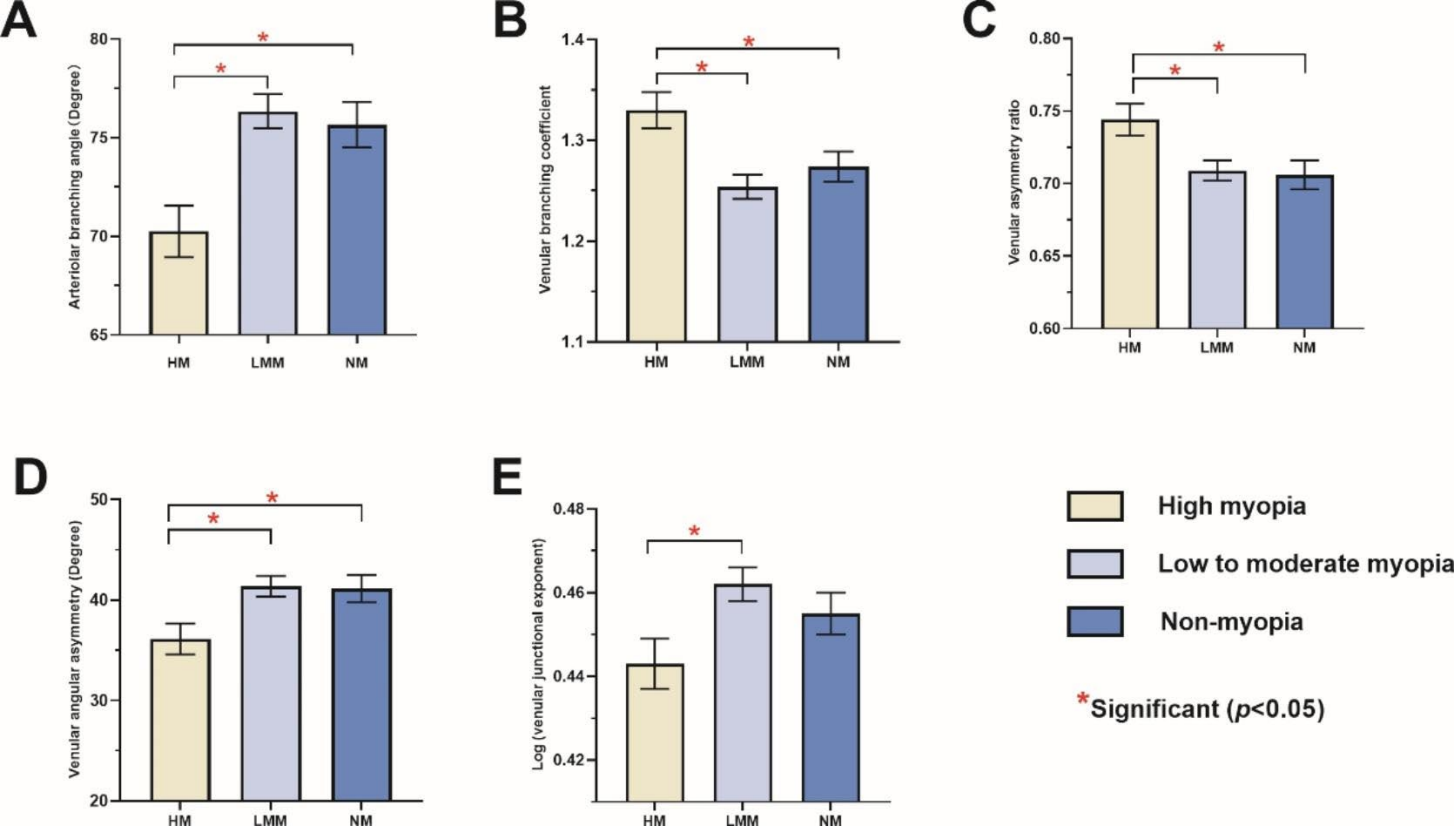
Bar charts showing the difference in the arteriolar branching angle (A), venular branching coefficient (B), venular asymmetry ratio (C), venular angular asymmetry (D), and log (venular junctional exponent deviation) (E) among high myopia, low to moderate myopia and non-myopia. Standard deviation bars are shown

The differences in the arteriolar branching angle, venular branching coefficient, venular asymmetry ratio, venular angular asymmetry and venular junctional exponent among three groups remained significant after adjusting for age, HbA1c, MAP, BMI and IOP (Table 3). Figure 2 shows arteriolar branching angle and venular angular asymmetry in high myopia were significantly lower than low to moderate myopia (*p* < 0.001, *p* = 0.014 respectively) and non-myopia (*p* = 0.007, *p* = 0.048 respectively). Venular asymmetry ratio and venular branching coefficient in high myopia were significantly higher than moderate myopia (*p* = 0.029, *p* = 0.001 respectively) and non-myopia (*p* = 0.041, *p* = 0.043 respectively). There was a significant difference in venular junctional exponent between the high myopia group and the low to moderate myopia group (*p* = 0.031). However, the difference of venular junctional exponent in non-myopia and high myopia were not statistically significant (*p* = 0.504).

**Table 3:** The comparison of the retinal vascular bifurcation measurements among participants with different refractive error statuses in multivariable-adjusted model

Parameter	High myopia Adjusted mean (95% CI)	Low to moderate myopia Adjusted mean (95% CI)	Non-myopia Adjusted mean (95% CI)	*P*
Arteriolar branching angle (degree)	70.21 (67.64, 72.78)	76.30 (74.60, 78.00)	75.75 (73.49, 78.02)	< 0.001*
Venular branching angle (degree)	74.47 (72.03, 76.92)	76.80 (75.17, 78.42)	76.13 (73.98, 78.28)	0.299
Arteriolar branching coefficient	1.46 (1.42, 1.51)	1.41 (1.39, 1.44)	1.46 (1.42, 1.50)	0.071
Venular branching coefficient	1.33 (1.30, 1.37)	1.25 (1.23, 1.28)	1.27 (1.24,1.30)	0.001*
Arteriolar asymmetry ratio	0.82 (0.81, 0.84)	0.82 (0.81, 0.83)	0.81 (0.80, 0.82)	0.321
Venular asymmetry ratio	0.74 (0.72, 0.77)	0.71 (0.69, 0.72)	0.71 (0.69, 0.73)	0.021*
Arteriolar angular asymmetry (degree)	30.60 (27.89, 33.31)	33.79 (31.99, 35.58)	33.14 (30.75, 35.53)	0.151
Venular angular asymmetry (degree)	36.10 (33.07, 39.13)	41.35 (39.34, 43.37)	41.19 (38.52, 43.85)	0.014*
Log (arteriolar junctional exponent)	0.41 (0.40, 0.43)	0.42 (0.42, 0.43)	0.42 (0.40, 0.43)	0.251
Log (venular junctional exponent)	0.44 (0.43, 0.46)	0.46 (0.45, 0.47)	0.46 (0.44, 0.47)	0.036*

CI, confidence interval; Model adjusted for age, HbA1c, MAP, BMI, and IOP. P-values were corrected for multiple comparisons using the Bonferroni method

*Statistical difference (*p* < 0.05) among participants with different refractive error statuses

**Fig. 3:**
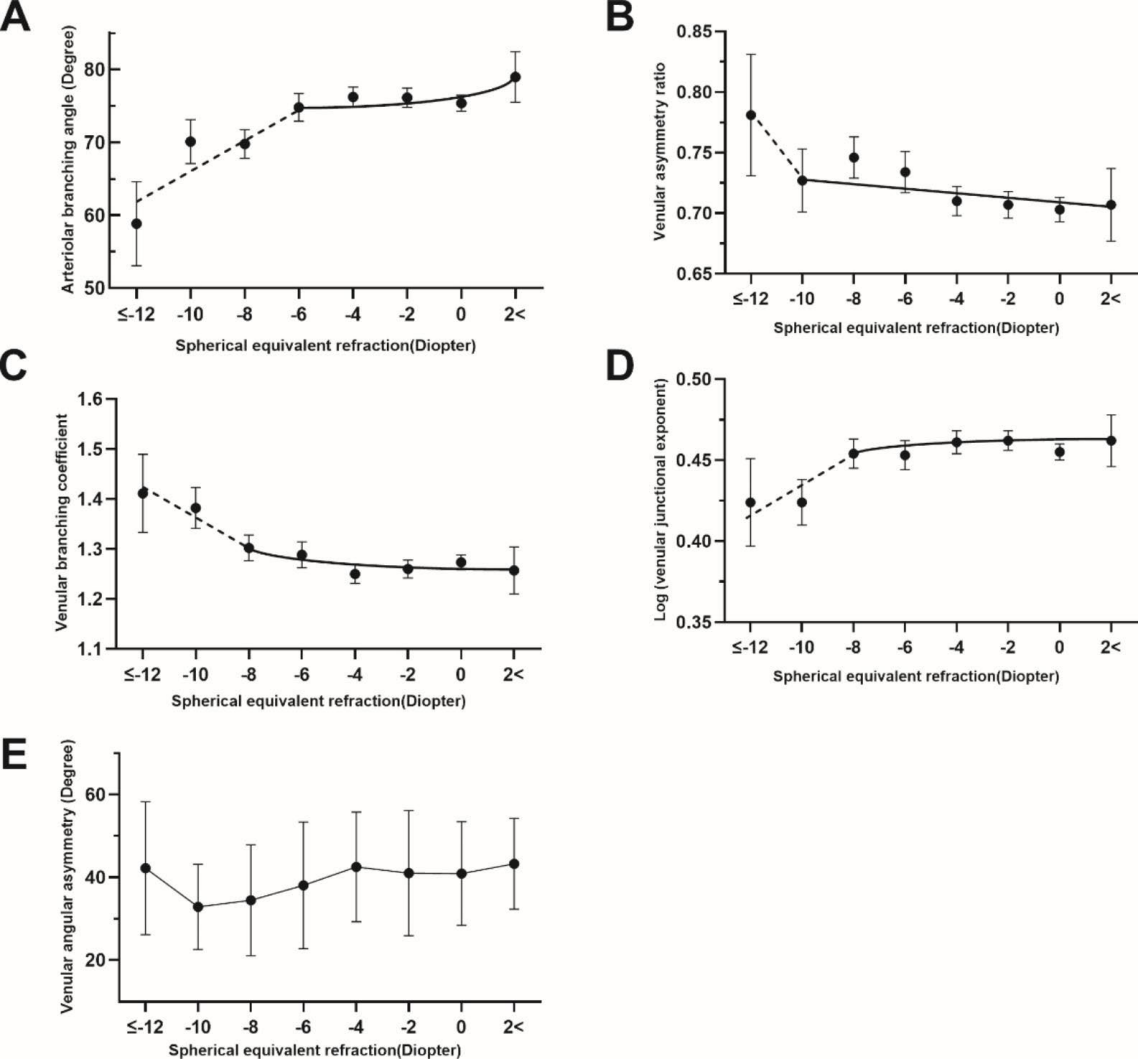
Arteriolar branching angle (A), venular asymmetry ratio (B), venular branching coefficient (C), log (venular junctional exponent deviation) (D), and venular angular asymmetry(E) distribution by spherical equivalent refraction (SER) after multivariable adjustment. SER was evenly divided into seven groups from − 12D to 2D. Mean value with 95% confidence interval (95% CI) was plotted against each refraction group

Figure 3 depicts the changes of arteriolar branching angle, venular asymmetry ratio, venular branching coefficient, venular junctional exponent, and venular asymmetry ratio with SER. It is observed that a sharp change of the slope occurred during the transition to higher myopia (roughly − 6D~-10D) in Fig. 3 (A ~ D) diagram. The slope for arteriolar branching angle by SER went steeper when SE ≤ -6D. For venular asymmetry ratio, venular branching coefficient, and venular junctional exponent, the cutoffs were − 10D, -8D, and − 8D, respectively. No clear cut-off point was observed for the chart of venular angular asymmetry (Fig. 3E).

A The slope for change in arteriolar branching angle by SER was steeper from − 6D. B The slope for change in venular asymmetry ratio by SER was steeper from − 10D. C The slope for change in venular branching coefficient by SER was steeper from − 8D. D The slope for change in log (venular junctional exponent deviation) by SER was steeper from − 8D.

## Discussion

Retinal vascular bifurcation parameters are potential markers of the changes in microvascular structure and could be early indicators of ocular and systemic disease [1, 5]. In the present study on an otherwise healthy and relatively young population, we first validated the association between retinal vascular bifurcation parameters and myopia; then we found a much significant change of the parameters in high myopia. The deviation from a so-called optimal mode of vascular architecture [22, 23], could potentially compromise the blood oxygen transport to the peripheral inner retina.

We observed that arteriolar branching angle was significantly smaller in the high myopia group than in low to moderate myopia group and non-myopia group. Compared with the low to moderate myopia and non-myopia, high myopia had a more acute branching angle (70.11°±11.73° versus 76.59°±11.41° versus 75.30°±10.66°, *p* < 0.001). Evidence has shown a distortion of retinal branching network hampered microcirculatory transport, increased shear stress, reduced efficiency, and hence greater risk of vascular damage [2].

Increased venular branching coefficient was significantly associated with high myopia in the current study. Branching coefficient increases when the diameter of the branching venules increases disproportionately to that of the parent vessel. The increase in the diameter of branching vessels may be due to vasodilation and result in thinning of the vessel wall. The thinner walls of branching venules may be less resistant to wall shear stress than parent venules, resulting in endothelial inflammation. It also has been suggested that an increased venular branching coefficient may reflect inflammation and endothelial dysfunction triggered by hyperglycaemia in diabetes [24].

Our findings indicate a relationship between decreased venular angular asymmetry and high myopia, and between increased venular asymmetry ratio and high myopia. It is of interest to evaluate the possible pathophysiological basis of these results. Although we are not aware of any studies that have investigated how varying angular asymmetry and asymmetry ratio would affect stress in the vasculature, and what effect these changes will have on the retina of high myopia. Some studies have reported that venular asymmetry ratio changes may precede Alzheimer’s disease. Cabrera et al. observed that the venular asymmetry ratio was significantly higher in patients with cognitive impairment than in age-matched controls [25]. Frost et al. observed venular asymmetry ratio was higher in healthy individuals with high plaque burden, that change may be occurring early in Alzheimer’s disease pathogenesis [26]. It is possible that increased venular asymmetry ratio was associated with microvascular damage.

An interesting observation was that repeatedly, high myopia has been associated with a lower risk for the development and progression of diabetic retinopathy (DR) [27]. The possible mechanism of the protective effect has not been fully elucidated, but may include decrease in retinal blood flow perfusion, decrease in retinal / choroidal oxygen demand, posterior vitreous detachment (PVD) and changes in cytokine profile in high myopia [27]. Some of the factors are independent of the changes of myopic vasculature, such as liquefication of vitreous humor / PVD in myopia [28]. Some other factors, however, could be the sequelae of the compromising blood flow transportation. Chronicle sub-optimal blood supply, among others, may lead to the degeneration or adaptation of retinal ganglion cells (RGCs) and retinal pigment epithelium (RPE) or other retinal cells and, in turn, decrease the oxygen demand [29]. As for the changes of the vascular geometric parameters in myopia, some may either be the causes or indicators of the protective effect of high myopia for DR. The narrowing of vascular caliber and the decrease of vascular fractal dimension (the complexity of vascular branching) in high myopia are related to the decrease of retinal blood flow [30]. Studies consistently showed that increased retinal blood flow volume is a significant risk factor for DR development or progression [27]. High flow volume may increase the physical stress to the retinal vascular wall, as well as the biochemical damage of hyperglycemia in diabetes. On the other hand, larger arteriolar or venular branching angle have been observed in diabetic or DR patients [5, 19]. It was also reported that larger arteriolar branching angle was frequently a concomitant of other risk factors, such as poor glycemic control [31] and longer diabetes duration [17]. Therefore, a reduced blood flow in myopia, indicated by narrower retinal vessels and simpler fractal dimension, and the smaller branching angles in high myopia, may lessen the damage and be protective. However, there is a lack of direct evidence of the causality. Further studies are needed.

A strength of the present study in comparison with previous reports is that we selected a more homogenous otherwise healthy population to minimize potential or residual confounding. Second, by studying the dose-effect of myopia on retinal vascular bifurcation parameters, we demonstrated that compared to the lower myopia, it seems that high myopia compromises the integrity of retinal vasculature at an accelerated rate. The finding of the current study contributes to the body of evidence about the interaction between myopia and retinal vessels – myopia stretches the globe as well as the domestic vascular system, while the latter in turn depletes the blood supply to the eye, which may lead to the anatomic and functional damage of high myopia, and play a role in the pathogenesis of pathologic myopia.

We acknowledge that some limitations affect the present study: first, the cross-sectional design limits the ability to infer causality. Second, the axial length was not measured in the current study. However, in a healthy and relatively young population, SER can be a very good proxy for the axial length [32]. Third, the number of high myopia participants in our study was insufficient for subgroup modeling.

In summary, the vascular bifurcation differs in dependence on the myopic refractive error and a significant increase in the difference can be observed in high myopic eyes. We believe that our study is the first to show the markedly deleterious effect of high myopia on retinal bifurcation. And if the finding is validated in future longitudinal study, it will deepen our understanding of the pathophysiology of the progression of myopia.

## Data Availability

The datasets generated and/or analyzed during the current study are not publicly available due further analysis of data is in progress, but are available from the corresponding author on reasonable request.
